# The Impact of Physical Exercise on Obesity in a Cohort of Southern Italian Obese Children: Improvement in Cardiovascular Risk and Immune System Biomarkers

**DOI:** 10.3390/ijerph20010602

**Published:** 2022-12-29

**Authors:** Cristina Mennitti, Annaluisa Ranieri, Ersilia Nigro, Lorella Tripodi, Mariarita Brancaccio, Jacopo Ulisse, Luca Gentile, Fabio Fimiani, Arturo Cesaro, Giovanni D’Alicandro, Giuseppe Limongelli, Aurora Daniele, Raffaela Pero, Giulia Frisso, Paolo Calabrò, Lucio Pastore, Maria Rosaria Licenziati, Olga Scudiero, Barbara Lombardo

**Affiliations:** 1 Department of Molecular Medicine and Medical Biotechnology, University of Naples Federico II, 80131 Naples, Italy; 2 Ceinge Biotecnologie Avanzate S. C. a R. L., 80131 Naples, Italy; 3 Department of Environmental, Biological and Pharmaceutical Sciences and Technologies (DISTABIF), University of Campania Luigi Vanvitelli, Via Vivaldi 43, 81100 Caserta, Italy; 4 Integrated Department of Laboratory and Transfusion Medicine, University of Naples Federico II, 80131 Naples, Italy; 5 Unit of Inherited and Rare Cardiovascular Diseases, A.O.R.N. Dei Colli “V. Monaldi”, Via Leonardo Bianchi, 80131 Naples, Italy; 6 Department of Translational Medical Sciences, University of Campania “Luigi Vanvitelli”, 80138 Napoli, Italy; 7 Division of Clinical Cardiology, A.O.R.N. “Sant’Anna e San Sebastiano”, 81100 Caserta, Italy; 8 Department of Neuroscience and Rehabilitation, Center of Sports Medicine and Disability, AORN, Santobono-Pausillipon, 80122 Naples, Italy; 9 Department of Cardio-Thoracic and Respiratory Sciences, University of Campania “Luigi Vanvitelli”, 80138 Napoli, Italy; 10 Task Force on Microbiome Studies, University of Naples Federico II, 80100 Naples, Italy; 11 Obesity and Endocrine Disease Unit, Department of Neuroscience, Santobono-Pausilipon Children’s Hospital, 80129 Naples, Italy

**Keywords:** childhood obesity, physical exercise, interleukins, defensins, cardiovascular risk, inflammation

## Abstract

Background: Childhood obesity (CO) is a serious medical condition affecting approximately 120 million children and adolescents worldwide. It is characterized by a persistent inflammatory state with inflammatory markers overexpressed, which in turn leads to a higher cardiovascular risk. It is well known that physical exercise reduces the inflammatory state in obese children. In the present study, we evaluated various biochemical parameters in obese children performing physical exercise compared to a group of obese sedentary children. Hence, the objective is to identify a panel of biomarkers to prevent numerous obesity-related complications. Methods: We examined two populations: 44 sedentary obese children (OSe), recruited on 5 November 2018 from Santobono–Pausilipon Children’s Hospital, Naples (Italy) of age = 11 ± 3.3 and 30 obese children who practice sport (OSp) of age = 10 ± 2.5. We observed a significant variation in some biochemical parameters such as white blood cells, C-reactive protein (CRP), glycemia and insulinemia. Moreover, we determined the levels of interleukins, chemokines and defensins by ELISA assay. Results: Our results showed a reduction in serum level of glycemia (*p*-value < 0.001), neutrophils (*p*-value < 0.05) and CRP (*p*-value < 0.05), whereas no relevant variations have been reported in insulin levels. Moreover, we found a decrease in serum levels of PDGF-β (*p*-value < 0.05), IL-9 (*p*-value < 0.01), IL-6 (*p*-value < 0.0001), IL-8 (*p*-value < 0.0001), IP-10 (*p*-value < 0.01), Eotaxin (*p*-value < 0.0001) and GM-CSF (*p*-value < 0.01) in OSp population in comparison to OSe. At the same time, we did not observe any significant variation in serum levels of IL-1ra and IL-17 between the two populations. On the other hand, we found an increase in HNP-1 (*p*-value < 0.0001) and HBD1 (*p*-value < 0.01) in OSp if compared to OSe. Conclusions: This study shed light on the role of physical exercise on CO, demonstrating in our population that an early evaluation of some biochemical parameters could be an assumption to prescribe physical exercise in order to monitor and prevent childhood obesity and related disorders.

## 1. Introduction

The World Health Organization (WHO) defines overweight and obesity as an accumulation of abnormal or excessive fat resulting in a risk to health and representing one of the most serious public health problems of this century [1]. Obesity is a pro-inflammatory state that increases the risk of several chronic diseases such as hypertension, dyslipidemia, type 2 diabetes, cardiovascular disease, asthma, osteoarthritis, infertility and increased risk of cancer, causing morbidity and mortality worldwide [2,3]. Smith JD et al. reported consistent data that pediatric obesity shows the same chronic diseases and risk factors that are identified in adults [4]. Childhood obesity is a complex disease characterized by having a body mass index (BMI) at or above the 95th percentile on the Centers for Disease Control and Prevention’s (CDC) specific growth charts. This pathological condition has several multifactorial causes: unmodifiable factors such as genetic characteristics, environmental factors and living conditions, as well as modifiable factors such as physical exercise, diet, sleep deprivation, parental determinants and socio-economic status [5,6,7]. In CO, the increase in adipose tissue results in weight gain and, consequently, in a persistent inflammatory state, with an alteration of the immune system. In most cases, lack of physical activity and unhealthy eating habits reinforce the occurrence of overweight and obesity in children and adolescents [8].

Lifestyle modification with increased intake of fruits and vegetables, along with recommended physical activity, form the mainstay for the primary treatment of childhood obesity [9]. This combined approach has been shown to promote weight loss and improve body composition, preserving muscle tissue and increasing fat loss [6]. Based on clinical research progress, in 2020, the WHO recommended performing an active lifestyle with appropriate levels of physical exercise needed to reduce the risk of preventable adverse health events for all people [10].

In this regard, several studies have shown that workout and exercising with obese children and adolescents has beneficial effects on symptoms of metabolic syndrome and low-grade systemic inflammation beyond the maintenance of body weight and improvement in the cardiorespiratory fitness level [11,12].

Studies conducted on obese children show that dysregulation of cytokine levels contributes to the pathogenesis of obesity and its related disorders [13,14,15,16,17,18,19]. This imbalance causes a persistent inflammatory state associated with obesity and the risk of developing complications related to obesity [3]. Specifically, increased levels of the neutrophil count, white blood cell, and C-reactive protein (CRP) were found to be positively associated with body mass index (BMI) and index of insulin resistance in obese children. Additionally, an increase in pro-inflammatory cytokines such as leptin, IL-6, and TNF-alpha was detected in overweight children compared to those of normal weight [20,21,22].

Physical activity modulates the production of cytokines in the obese, causing a beneficial effect in the prevention of comorbidities in obese subjects and improving the quality of life [23,24,25]. IL-6 is a pro-inflammatory cytokine involved in the regulation of the inflammatory response and controls CRP synthesis in the liver. Increased IL-6 levels in obese patients may increase the risk of developing cardiovascular complications, insulin resistance and type 2 diabetes. Therefore, a decrease in body weight in individuals who play sports reduces IL-6 levels and, consequently, CRP levels due to less fat being available. Low CRP levels result in low permeability of the vascular wall to LDL, thus reducing the risk of cardiovascular disease (CVD) [26]. IL-8 and IL-9 are pro-inflammatory cytokines implicated in the pathogenesis of atherosclerosis, with an increased risk of developing cardiovascular disease [27,28,29]. Studies show that exercise involves a decrease in plasma levels of IL-8 and IL-9, with a protective anti-inflammatory effect of exercise compared to atherosclerotic risk [30,31]. Furthermore, among the anti-inflammatory cytokines, the serum concentration of the IL-1Ra in obese children seems to play a role in reducing susceptibility to type 1 and type 2 diabetes, representing an important marker of inflammatory response linked to obesity in the pediatric population [32,33,34,35]. The GM-CSF is a pro-inflammatory cytokine involved in the recruitment and activation of macrophages in adipose tissue and appears to play an important role in the overall expression levels of pro-inflammatory cytokines in adipose tissue [36]. Eotaxin is an important eosinophilia-specific chemokine, which is associated with the recruitment of eosinophils to sites of inflammation and eosinophils activated may release a range of potent immuno-modulatory factors, including cytokines, chemokines, and growth factors, which have a marked effect on the progression of immune and inflammatory responses [37,38]. The IP-10 is significantly higher in obese subjects and is significantly associated with the degree of BMI and obesity and the HOMA-IR index, indicating their potential as biomarkers of insulin resistance. IP10 may play a deleterious role in obesity as a potential inhibitor of adipose tissue angiogenesis. High levels of IP10 could lead to insufficient angiogenesis, which has been associated with chemotaxis and inflammation of leukocytes in fat stores, contributing to the transition to metabolic dysfunction in obesity [39]. Furthermore, PDGF-β is involved in the development of adult obesity; in fact, it promotes the angiogenesis of adipose tissue, which is responsible for the expansion of tissues in obesity [40]. The reduction of the levels of IL-6, IL-8 and IL-9 in obese subjects who play sports shows the beneficial effect of physical activity in modulating the systemic inflammation underlying obesity and the potential contribution to the prevention of its complications. The decrease in levels of GM-CSF, Eotaxin, IP10 and PDGF-β, found in the group of obese children who play sports compared to sedentary obese children, could be further investigated in order to identify new markers of the inflammatory state. Finally, a key role in the immune system is played by the defensins, a significant family of antimicrobial peptides (AMPs) which are involved in infection and inflammation [41,42]. Human defensins are divided into two groups: alpha and beta-defensins, showing antimicrobial activity and multiple roles in innate immunity. In particular, it has been found that HPN1 and HBD1 levels increase in individuals who practice physical activity [43,44].

Based on this evidence, we can assert that regular physical activity reduces the risk of developing CVDs, type 2 diabetes mellitus, risk of developing cancer, depression, anxiety and other diseases and, in some cases, the therapeutic effects related to physical activity may be comparable or superior to those of standard drug therapies [45]. So, physical exercise represents a non-pharmacological strategy for attenuating obesity-related complications, which in turn limits the impact on long-term health and society [46,47].

To date, recent studies focused on basal parameters of laboratory medicine such as leukocytes, lymphocytes and neutrophils counts, CRP and some cytokines [48,49]. We hypothesized that a broad analysis including more biochemical parameters and inflammatory markers such as cytokines, chemokines and defensins profile could contribute to a better and deep evaluation of low-grade inflammation and complications related to obesity. We retain that an extensive analysis of laboratory medicine could offer a real chance to treat and prevent childhood obesity, protecting their health now and in the future.

In the present cross-sectional study, we identified a novel specific panel of markers of cardiovascular risk and pro/anti-inflammatory immune system to monitor the beneficial effects of physical activity in obese children. This active surveillance may prevent several obesity-related complications and may lead to an early identification of risk factors for childhood obesity in order to set up an adequate and specific therapeutic approach.

## 2. Materials and Methods

### 2.1. Ethical Approval

The study was conducted according to the ethical guidelines of the Helsinki Declaration of the World Medical Association and was approved by the ethics committee (protocol 101/21) of the University of Naples Federico II.

### 2.2. Recruitment and Participants

For this study, we recruited 74 male children: 44 sedentary obese children (OSe) and 30 obese children who practiced sport (OSp), from Santobono–Pausilipon Children’s Hospital and collected peripheral blood from both obese populations. All subjects were informed of the purpose and procedures of the study, and written informed consent was obtained from each parent, being a participant under 18. In Table 1, inclusions and exclusion criteria have been reported. In particular, we have included children with BMI equal to or greater than the 95th percentile, and for this reason, they would be considered as having obesity according to CDC Growth Charts [50].

Moreover, parents of the recruited children were subjected to a survey to investigate their children’s eating habits, and it was found that none of them followed a food restriction at the time of the investigation.

Obese children who play sports are involved in the following activities for about 12 months: football, swimming, basketball, and karate. Anthropometric characteristics of obese children are reported in Table 2.

### 2.3. Treadmill Stress Test

Sedentary obese children and obese children who practice sports performed treadmill stress testing. First of all, resting ECG, heart rate, and blood pressure were obtained prior to starting the exercise regimen. The resting ECG has been obtained both supine and standing since the patient’s position could influence the QRS and T wave axes. Then, electrodes were placed on the chest and attached to an ECG machine, recording the heart’s electrical activity. Every child was placed on a treadmill, monitoring for any developing symptoms such as chest pain, shortness of breath, dizziness, or extreme fatigue. Each child performed the treadmill stress test in the morning, followed a 3-h fast, and ran for 15-min on the treadmill at an increasing speed and gradient to assess any changes in the electrocardiograph and monitor blood pressure. All the children completed the test without interruption. Moreover, patients were questioned about any symptoms they experienced during exercise. All patients were monitored closely during recovery until heart rate and ECG were back to baseline, as arrhythmias and ECG changes could still develop.

### 2.4. Biochemical Determinations

Blood samples were taken in the morning (8:00 a.m.) for all children after 12 h of rest. Each participant was subjected to a blood sample.

White blood cell counts were performed using the Siemens Advia 2120i hematology analyzer (Siemens Healthcare, Munich, Germany) according to the manufacturer’s recommendations. The level of CRP was evaluated on Architect c16000 (Abbott Diagnostics, Chicago, IL, USA) according to the manufacturer’s recommendations.

All analysis was performed in duplicate to guarantee the accuracy of results.

### 2.5. Evaluation of Glycemia and Insulinemia in Obese Pediatric Population

All patients performed an Oral Glucose Tolerance Test (OGTT) to determine glycemia and insulinemia concentrations. Blood samples were taken in the morning when the circadian rhythm of glucose metabolism was highest. Patients fasted for 8 h prior to the test but maintained their typical diet for the previous days. OGTT is divided into several phases:

1. A first baseline sample is taken in the morning at 8:00 a.m. (time zero T0).
2. The patient drinks a solution containing 75 g of glucose dissolved in 300–500 mL of water. The solution must be swallowed in a maximum time of 5 min.
3. After 120 min from T0, we collected a second blood sample (T 120). In our case, the OGTT is called “two-stroke”.

The levels of glycemia were evaluated on Architect c16000 (Abbott Diagnostics, Chicago, IL, USA) according to the manufacturer’s recommendations. Insulinemia has been performed on ADVIA Centaur (Siemens Healthcare, Munich, Germany) according to the manufacturer’s recommendations.

### 2.6. Elisa Assay

PDGF-β, GM-CSF, IP-10, Eotaxin and interleukins (IL-1ra, IL-9, IL-17, IL-6 and IL-8) were measured by Bio-Plex Multiplex immunoassays (Bio-Rad, Hercules, CA, USA) according to the manufacturer’s recommendations. The Bio-Plex Multiplex immunoassays allow us to quantify multiple protein biomarkers in a single well of a 96-well plate in 3–4 h from just 12.5 μL of serum or plasma. In this assay, capture antibodies directed against the desired biomarker are covalently coupled to the beads. The paired beads react with the sample containing the biomarker of interest. After a series of washes to remove unbound proteins, a biotinylated sensing antibody is added to create a sandwich complex. The final sensing complex is formed with the addition of the streptavidin-phycoerythrin conjugate (SA-PE). Phycoerythrin serves as a fluorescent indicator or reporter. Reaction data is acquired using a Bio-Plex system where a red laser (635 nm) illuminates the fluorescent dyes within each bead to provide bead grading, and a green laser (532 nm) excites PE to generate a reporter signal, which is detected by a photomultiplier tube (PMT). The Bio-Plex Manager software presents data as median fluorescence intensity (MFI) and concentration (pg/mL). The concentration of analyte bound to each bead is proportional to the MFI of the reporter signal. Then, using Bio-Plex Data Pro software, data from multiple instrument runs can be combined into a single project for quick results visualization and simple statistical analysis.

Alpha-defensin 1 and beta-defensin 1 were assessed in the serum using ELISA (Human DEFα1 and Human DEFB1 ELISA Kit, Elabscience, Buckingham, UK) according to the manufacturer’s recommendations. The ELISA kit uses the Sandwich-ELISA principle. The micro-ELISA plate has been pre-coated with an antibody specific to specific human defensin. Samples are added to the micro-ELISA plate wells and combined with the specific antibody. Then a biotinylated detection antibody specific for human defensin and Avidin-Horseradish Peroxidase (HRP) conjugate are added successively to each microplate well and incubated. Free components are washed away. The substrate solution is added to each well. Only those wells that contain human defensins, biotinylated detection antibodies and Avidin-HRP conjugate will appear blue in color. The enzyme-substrate reaction is terminated by the addition of a stop solution, and the color turns yellow. The optical density (OD) is measured spectrophotometrically at a wavelength of 450 nm ± 2 nm. The OD value is proportional to the concentration of human defensin. The concentration of human defensin in the samples can be calculated by comparing the OD of the samples to the standard curve.

All analysis was performed in triplicate to guarantee the accuracy of results.

### 2.7. Data Analysis and Statistics

All statistical analyses were performed using GraphPad Prism 8.4.0 (GraphPad Software Inc., La Jolla, CA, USA). Data were expressed as the means ± standard deviations. Unpaired Student’s *t*-test was used to compare the groups, with values of *p* < 0.05 considered significant. This test is one of the most popular statistical techniques used to test whether the mean difference between two groups is statistically significant.

## 3. Results

### 3.1. Impact of Physical Activity on Cardiac Parameters in Childhood Obesity

In order to evaluate the effect of physical activity on childhood obesity, sedentary obese children and obese children who practiced sports performed a treadmill stress test during which the following parameters were analyzed: blood pressure and heart rate (HR) before and after exercise and the maximum volume of oxygen (VO2max) after exercise. The results obtained before and after the stress test are shown in Table 3.

As shown in Table 3, it was observed that diastolic blood pressure is slightly higher in sedentary obese children (69.6 ± 7.6 mmHg) than in obese who played sports (68.4 ± 7.3 mmHg), but no significant changes were observed between the two groups. The same is verified for systolic blood pressure, which is slightly higher in sedentary obese children (110.2 ± 10.1 mmHg) than in sports obese (108.9 ± 9.1 mmHg). Despite these notes, we can say that, before exercise, the blood pressure in OSe and OSp remains within the normal range (80–120 mmHg). Resting HR was then calculated: the sports obese have a decrease in HR compared to the sedentary obese (*p* value <0.001) in fact, we have 82 beats/min in OSe and 72 beats/min in OSp before exercise; both values are normal (infant population: 70–100 beats/min).

The same parameters were evaluated after physical exertion: the blood pressure did not show significant differences between the two groups, but the same parameter, observed after the test, slightly increased; in fact, in both populations, it was equal to 128 mmHg. After the stress test, HR was equal to 163.4 ± 15.1 beats/min in OSe and 163.8 ± 16.3 beats/min in the OSp, and consequently, under exertion, there was no significant change in HR. The load evaluated during the stress test in the two populations was 79.0 ± 21.1 W in OSe and 83.3 ± 18.3 W in OSp, showing a slight and significant decrease in the load for the group that did not practice sports (*p*-value < 0.0474).

Finally, following the stress test, the VO2max was also evaluated, which showed no variation in the two study groups: 37 mL/kg/min for the OSe and 38 mL/kg/min for OSp.

### 3.2. Effect of Physical Activity on the Immune System of Obese Children

**Figure 1:**
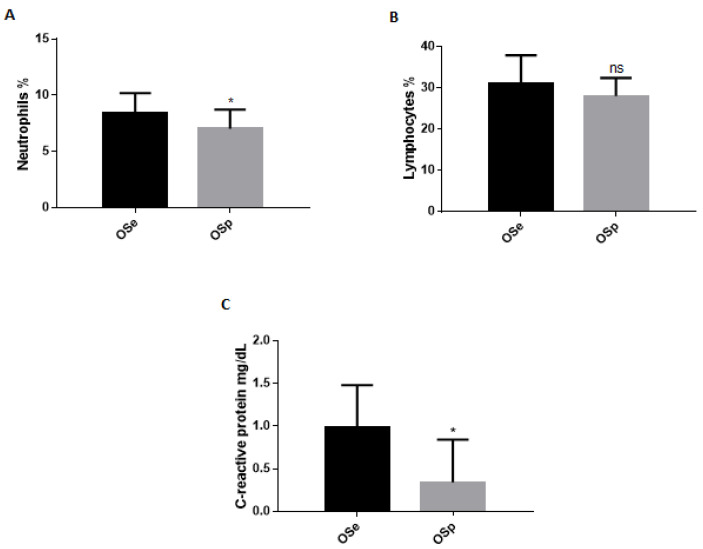
White blood cells and C-reactive protein in sedentary obese children (OSe) compared to obese children who played sport (OSp). (**A**) Neutrophils, (**B**) Lymphocytes, (**C**) C-reactive protein. The data are expressed as the means ± SDs. The significance was determined by the Student’s *t*-test: * (*p* < 0.05), which represents significance compared to sedentary obese children, ns (*p* > 0.05), not significative compared to sedentary obese children.

To evaluate the immune health status of obese children, we assessed white blood cells (Figure 1A,B). In addition, we determined the levels of CRP (Figure 1C) to evaluate the severity of the inflammatory state caused by obesity. Our results have shown a significant decrease in both the percentage of neutrophils and CRP levels in obese children who practiced sports compared with sedentary obese children (Figure 1A,C). Meanwhile, no significant difference in the percentage of lymphocytes was observed between the two groups (Figure 1B).

### 3.3. Determination of Glycemia and Insulinemia in Obese Children Population

**Figure 2:**
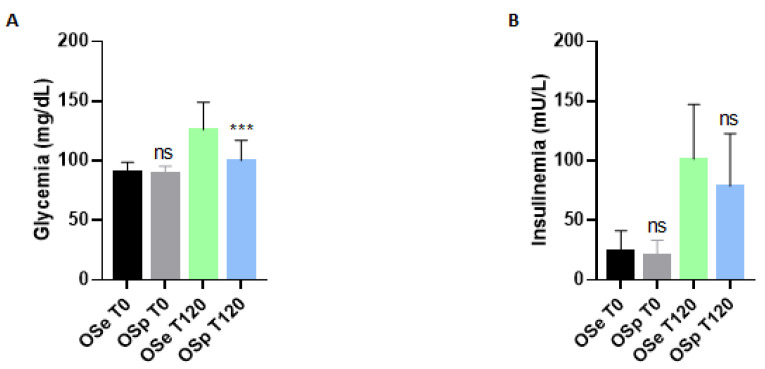
Determination of glycemia and insulin levels in sedentary obese children (OSe) compared to obese children who played sport (OSp). (**A**) Glycemia, (**B**) Insulin. Data are expressed as the means ± SDs. The significance was determined by the Student’s *t*-test: *** (*p*-value < 0.001) and ns (*p* > 0.05), not significative compared to sedentary obese children.

A classic OGTT was performed for the evaluation of oral glucose tolerance. In our case, we chose to compare only two times: the basal sampling (T0) and after 2 h 120’ (T120). The results are shown in Figure 2.

As for glycemia, there was no statistically significant variation at T0 between the two populations. In fact, values were almost similar. Conversely, after oral glucose loading, a notable difference emerged at T120 between the two groups: sedentary obese children (OSe) showed a higher glycemia (126 ± 23.4 mg/dL) than the population of obese children who played sports (OSp), which had a lower glycemia (100.2 ± 16.1 mg/dL) (Figure 2A).

Figure 2B represents insulinemia levels in obese children. Results showed that variations were not statistically significant at T0, and the same trend has been observed in T120, but underlying a better reduction in OSp a T120 (78.73 ± 44.21 mU/L).

### 3.4. Evaluation of Interleukins Level in Obese Children

**Figure 3:**
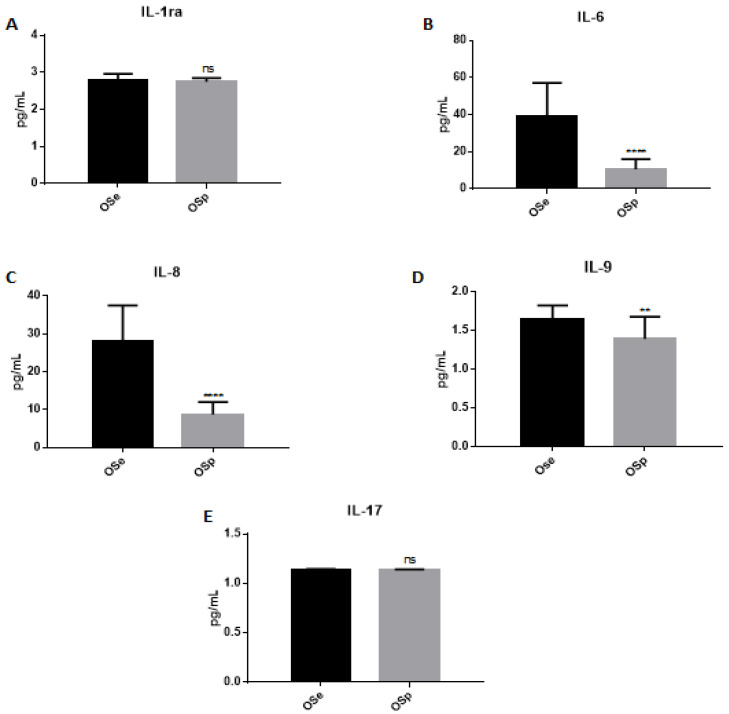
Evaluation of interleukins level in the serum of sedentary obese children (OSe) compared to obese children who played sport (OSp). (**A**) IL-1ra, (**B**) IL-6, (**C**) IL-8, (**D**) IL-9, (**E**) IL-17. The data are expressed as the means ± SDs. The significance was determined by the Student’s *t*-test: **** (*p* < 0.0001) and ** (*p* < 0.01) represent significance compared to sedentary obese children, ns (*p* > 0.05), not significative compared to sedentary obese children.

To understand whether physical activity could positively influence the inflammatory state caused by obesity within the two populations under study, we evaluated the levels of some pro-inflammatory interleukins. In particular, as shown in Figure 3, we did not observe a significant difference between IL-1ra and IL-17 (Figure 3A,E). At the same time, there is a significant decrease in the levels of IL-6, IL-8 and IL-9 in the obese physically active population compared to the sedentary obese (Figure 3B, Figure 3C and Figure 3D, respectively).

**Figure 4:**
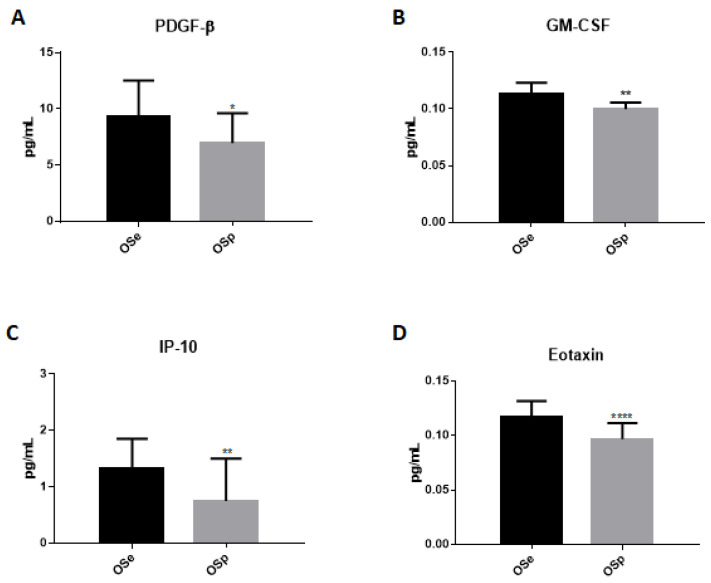
Dosage of cytokines and chemokines in the serum of sedentary obese children (OSe) compared to obese children who played sport (OSp). (**A**) PDGF-β, (**B**) GM-CSF, (**C**) IP-10, (**D**) Eotaxin. The data are expressed as the means ± SDs. The significance was determined by the Student’s *t*-test: * (*p* < 0.05), ** (*p* < 0.01) and **** (*p* < 0.0001) represent significance compared to sedentary obese children.

In addition, we evaluated the serum levels of some chemokines and cytokines (Figure 4) in order to understand if physical activity could be a protective factor against inflammation related to obesity. Data obtained show that the levels of PDGF-β, GM-CSF, IP-10, and Eotaxin significantly decreased in the obese population who practiced sports when compared with the sedentary obese (Figure 4A–D).

### 3.5. Dosage of HNP-1 and HBD-1 in Childhood Obesity

**Figure 5:**
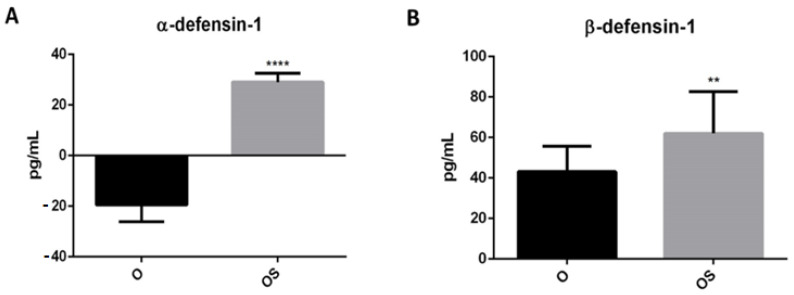
Accumulation of defensins in serum of sedentary obese children (OSe) compared to obese children who played sport (OSp). (**A**) α-defensin-1 and (**B**) β-defensin-1. The data are expressed as the means ± SDs. The significance was determined by the Student’s *t*-test: ** (*p* < 0.01) and **** (*p* < 0.0001), which represent significance compared to sedentary obese children.

To evaluate whether physical activity could influence the release of antimicrobial peptides, we measured HNP-1 and HBD-1 by ELISA assay. Our results showed that levels of both are higher in the population of obese children who practiced sports (Figure 5A,B). In particular, HNP-1 was not expressed in OSe (Figure 5A). In fact, a 1.5 times increase in expression can be observed in OSp (*p* < 0.0001). We observed a similar trend also for HBD-1, with a 1.5 times increase of expression in the OSp when compared with OSe (Figure 5B) (*p* < 0.01).

## 4. Discussion

Childhood obesity is a pathologic process characterized by a chronic inflammatory condition, and some of the comorbidities and risks related to excess weight may be associated with inflammatory aspects of the disease [51]. In particular, obese subjects show changes in systemic levels of several cytokines and increased concentrations of CRP, WBC and defensins [52]. Physical activity is able to induce biochemical changes lowering CRP levels, white blood cell number and systemic levels of inflammatory cytokines, a condition necessary to be protected against chronic disorders associated with low-grade systemic inflammation [53]. In this scenario, in our study, we have evaluated the effects of physical activity in an obese pediatric population in order to highlight possible changes and improvements in children who practice sports compared to sedentary obese children. Additionally, compared to previous studies [48,49], we consider a deep panel including more biochemical parameters and inflammatory markers.

Various factors can affect heart rate variability in individuals with obesity, including comorbidities, eating habits, physical activity, emotional stress, and genetic factors. In fact, it has been shown that individuals who are overweight have a sympathetic vagal imbalance due to the increase in sympathetic activity associated with visceral fat [16]. For this purpose, all obese children performed a treadmill stress test in order to evaluate the response of the cardiovascular and respiratory systems to exercise.

Obesity is often associated with insulin resistance (IR), a condition characterized by a reduced response of the tissues to insulin-mediated actions. In obese children and adolescents, IR and a higher prevalence of components of metabolic syndrome (MS) have been observed, which exposes subjects to higher cardiovascular risk. The main cause was found in the β cell dysfunction that occurs in obese children and adolescents due to increased fat storage [54]. For this reason, our obese populations have performed an OGTT in order to evaluate blood glucose and insulin levels in basal conditions and after glucose intake. From the results of OGTT, it emerged that, although there is not a noticeable change in T0, at T120, the pediatric population practicing sports had significantly lower blood glucose (100.2 ± 6.6 mg/dL) compared to the group of children who do not play sports (126 ± 23.4 mg/dL). The reference values of glycemia in the pediatric population are 60–100 mg/dL. Therefore, it is evident that the group that does not practice sports has glycemic values that exceed the normal range. Furthermore, excessively high blood glucose values at T120 are considered indicative of a possible pre-diabetes condition. On the other hand, no relevant variations have been observed in insulinemia in both populations. It is evident that the practice of sporting activity is always capable of inducing beneficial effects. Moreover, in the case of conditions such as obesity, it allows the modification of body composition with an improvement in fat deposits and an increase in muscle mass. Also, physical activity ameliorates capillary perfusion of muscles and the number of mitochondria, metabolic pathways involved in glycolysis and lipolysis, and intra-cellular transport of glucose by activation of GLUT-4 with increased insulin sensitivity and therefore determines a reduction in blood sugar [55].

In order to carry out an assessment of the inflammatory state in the two groups of our study, the dosage of some biochemical parameters such as WBC and CRP was performed. CRP is an acute-phase inflammatory protein that increases in obese patients and represents a marker for the early diagnosis of metabolic syndrome and cardiovascular risk in obese children [56]. Studies show that regular training induces a reduction in CRP levels suggesting that physical activity can suppress low-grade systemic inflammation, reducing the risk of cardiometabolic disorders and contributing to an improvement in quality of life [32]. An elevated WBC number found in obese patients is an important parameter of the inflammatory condition associated with obesity. More recently, features of metabolic syndrome, coronary artery disease and complications of type 2 diabetes have also been associated with higher WBC counts. In particular, a high number of WBC is a strong risk factor for CHD morbidity and mortality [57]. Physical activity appears to be effective in correcting the total leukocyte count by lowering the number of lymphocytes, monocytes and basophils and significantly reducing the factors related to CHD risk. A reduction in BMI by physical activity has been associated with a decrease in white blood cell numbers. For this reason, total WBC and neutrophil counts could be a potential parameter to the effectiveness of exercise in correcting low-grade systemic inflammation [58]. The evaluation of the biochemical parameters in the two different groups in our study showed a significant reduction in the percentage of neutrophils and CRP levels in OSp compared to OSe. This highlights an act of physical activity on inflammatory parameters related to the cardiovascular risk that could help to clarify the potential therapeutic effect of physical activity on low-grade systemic inflammation. Subsequently, the variations of some inflammatory cytokines levels in the two different groups were evaluated; a significant decrease was observed in the levels of IL-6, IL-8, IL-9, PDGF-β, GM-CSF, IP-10 and Eotaxin in the physically active obese population versus the sedentary obese population. There were no significant differences in the values of IL-1Ra and IL-17 between the two groups. Our results show an increase in defensin levels, stimulated by physical activity that favors its expression, which acts by increasing the body’s immune defenses. In particular, as shown in recent studies and in accordance with the data obtained in our study, serum increases in defensins α and β after regular physical activity could contribute to normalizing the levels of inflammation-related biochemical parameters such as CRP and white blood cell count.

## 5. Limitations and Future Prospects

The limitations of our study are mainly based on the small number of patients recruited. In fact, further studies performed on a larger population should be conducted. In addition, another limitation concerns the information relating to eating habits and energy intake coming from children’s responses questionnaire. Some answers may be conditioned by social prejudices. Furthermore, the education levels and socio-economic status of families were not evaluated.

Since childhood obesity is due to low-grade inflammation, we encountered difficulties in measuring some inflammatory cytokines. More sensitive techniques would be needed to detect even small increases in some cytokines.

In future studies, we plan to evaluate the effectiveness of a planned pattern of physical activity for obese children using a prospective research design and also to examine the relationships between nutrition, exercise, medical conditions, and childhood obesity.

## 6. Conclusions

An extensive analysis of biochemical parameters and inflammatory markers could contribute to a better evaluation of low-grade inflammation and complications related to childhood obesity. This continuous monitoring of laboratory medicine parameters, combined with the practice of physical exercise, could represent an opportunity to treat and prevent childhood obesity. In fact, regular sports practice might improve body composition, decrease metabolic complications, or enhance psychological profiles in children with obesity.

Incorporating sport as part of anti-obesity strategies in children and adolescents could represent a valid tool to safeguard the health of obese children, protecting them from the risks related to obesity. Finally, this strategy could allow the identification of new biomarkers and continuous monitoring could highlight the beneficial effects of physical activity in the prevention of complications and health-related quality of life. Further studies would be necessary to understand the benefits and role of physical activity as a powerful non-pharmacological tool against obesity in the pediatric age.

## Figures and Tables

**Table 1:** Inclusion and exclusion criteria.

Inclusion Criteria	Exclusion Criteria
BMI ≥ 95th percentile	Considerable mental or physical disabilities
Age ≥ 7.5	Non-metabolic comorbidities
Sex = male	Walking or gym practice
Southern Italian children	Drug addiction
Written informed consent by participants	

**Table 2:** Characteristics of the obese population. Anthropometric characteristics of children are expressed as mean ± SD.

Characteristics	OSe (*n* = 44)	OSp (*n* = 30)	*p* Value (<0.05)
age (in years)	11 ± 3.3	10 ± 2.5	0.287
weight (kg)	70 ± 23.3	61 ± 17.3	0.173
height (m)	1 ± 0.27	1 ± 0.13	>1
BMI	31 ± 6.9	28 ± 4.4	0.109
physical exercise (days a week)		2.5 ± 0.7	

**Table 3:** Assessment of VO2 max, blood pressure and heart rate in OSe and OSp before and after treadmill stress test.

Parameters and Reference Values	OSe	OSp	*p* Value (<0.05)
Blood Pressure before treadmill stress test (80–120 mmHg)	69.6 ± 7.6 110.2 ± 10.1	68.4 ± 7.3 108.9 ± 9.1	0.147 0.222
Heart Rate before treadmill stress test (70–100 beats/min)	82 beats/min	72 beats/min	0.001
VO2max after exercise stress test (Poor < 30; Excellent >48 mL/kg/min)	37 mL/kg/min	38 mL/kg/min	0.348
Blood Pressure after exercise stress test (80–120 mmHg)	128 mmHg	128 mmHg	>1
Heart Rate after exercise stress test (70–100 beats/min)	163.4 ± 15.1 beats/min	163.8 ± 16.3 beats/min	0.819

## Data Availability

All the results obtained from the study were reported in the manuscript. There is no additional data.

## Abbreviations

CO	Childhood obesity
OSe	Sedentary obese children
OSp	Obese children who practice sport
CRP)	C-reactive protein
ELISA	Enzyme-linked immunosorbent assay
PDGF-β	Platelet-derived growth factor subunit b
IL-9	Interleukin-9
IL-6	Interleukin-6
IL-8	Interleukin-8
IP-10	Human interferon-inducible protein 10
GM-CSF	Granulocyte-macrophage colony-stimulating factor
IL-1ra	Interleukin-1 receptor antagonist
IL-17	Interleukin-17
HNP-1	Human neutrophil peptide-1
HBD1	Human beta defensin-1
WHO	World Health Organization
BMI	Body mass index
CDC	Centers for Disease Control and Prevention
TNF-alpha	Tumor necrosis factor
LDL	Low-density lipoproteins
CVD	Cardiovascular disease
HOMA-IR	Homeostatic Model Assessment for Insulin Resistance
AMPs	Antimicrobial peptides
ECG	Electrocardiography
OGTT	Oral glucose tolerance test
SA-PE	Streptavidin-phycoerythrin conjugate
PMT	Photomultiplier tube
MFI)	Median fluorescence intensity
HRP	Horseradish peroxidase
OD	Optical density
HR	Heart rate
VO2max	Maximum volume of oxygen
WBC	White blood cells
IR	Insulin resistance
GLUT-4	Glucose transporter 4
CHD	Coronary heart disease

## References

[1] Güngör N.K. (2014). Overweight and obesity in children and adolescents. J. Clin. Res. Pediatr. Endocrinol..

[2] Kumar S., Kelly A.S. (2017). Review of Childhood Obesity: From Epidemiology, Etiology, and Comorbidities to Clinical Assessment and Treatment. Mayo Clin. Proc..

[3] Scudiero O., Pero R., Ranieri A., Terracciano D., Fimiani F., Cesaro A., Gentile L., Leggiero E., Laneri S., Moscarella E. (2019). Childhood obesity: An overview of laboratory medicine, exercise and microbiome. Clin. Chem. Lab. Med..

[4] Smith J.D., Fu E., Kobayashi M. (2020). Prevention and Management of Childhood Obesity and its Psychological and Health Comorbidities. Annu. Rev. Clin. Psychol..

[5] Ang Y.N., Wee B.S., Poh B.K., Ismail M.N. (2013). Multifactorial Influences of Childhood Obesity. Curr. Obes. Rep..

[6] Lee J.H., Cho J. (2022). Sleep and Obesity. Sleep Med. Clin..

[7] Ling C., Ronn T. (2019). Epigenetics in Human Obesity and Type 2 Diabetes. Cell Metab..

[8] Kansra A.R., Lakkunarajah S., Jay García-Hermoso M.S. (2021). Childhood and Adolescent Obesity: A Review. Front. Pediatr..

[9] Pandita A., Sharma D., Pandita D., Pawar S., Tariq M., Kaul A. (2016). Childhood obesity: Prevention is better than cure. Diabetes Metab. Syndr. Obes..

[10] Juonala M., Magnussen C.G., Berenson G.S., Venn A., Burns T.L., Sabin M.A., Srinivasan S.R., Daniels S.R., Davis P.H., Chen W. (2011). Childhood adiposity, adult adiposity, and cardiovascular risk factors. N. Engl. J. Med..

[11] Jakicic J.M., Davis K.K. (2011). Obesity and physical activity. Psychiatr. Clin. N. Am..

[12] Fang X., Henao-Mejia J., Henrickson S.E. (2020). Obesity and immune status in children. Curr. Opin Pediatr..

[13] Conlon K.C., Miljkovic M.D., Waldmann T.A. (2019). Cytokines in the Treatment of Cancer. J. Interferon Cytokine Res..

[14] Coppack S.W. (2001). Pro-inflammatory cytokines and adipose tissue. Proc. Nutr. Soc..

[15] Tayal V., Kalra B.S. (2008). Cytokines and anti-cytokines as therapeutics—An update. Eur. J. Pharmacol..

[16] Aygun A.D., Gundor S., Ustundag B., Gurgoze M.K., Sen Y. (2005). Proinflammatory cytokines and leptin are increased in serum of prepubertal obese children. Mediat. Inflam..

[17] Caballero A.E., Bousquet-Santos K., Robles Osorio L., Montagnani V., Soodini G., Porramatikul S., Hamdy O., Nobrega A.C.L., Horton E.S. (2008). Overweight Latino children and adolescents have marked endothelial dysfunction and subclinical vascular inflammation in association with excess body fat and insulin resistance. Diabetes Care.

[18] Sacheck J. (2008). Pediatric obesity: An inflammatory condition?. J. Parenter. Enter. Nutr..

[19] Parrett A.L., Valentine R.J., Arngrimsson S.A., Castelli D.M., Evans E.M. (2010). Adiposity, activity, fitness, and C-reactive protein in children. Med. Sci. Sports Exerc..

[20] Vehapoglu A., Turkmen S., Goknar N., Özer Ö.F. (2016). Reduced antioxidant capacity and increased subclinical inflammation markers in prepubescent obese children and their relationship with nutritional markers and metabolic parameters. Redox Rep..

[21] Murdolo G., Nowotny B., Celi F., Donati M., Bini V., Papi F., Gornitzka G., Castellani S., Roden M., Falorni A. (2011). Inflammatory adipokines, high molecular weight adiponectin, and insulin resistance: A population-based survey in prepubertal schoolchildren. PLoS ONE.

[22] Carolan E., Hogan A.E., Corrigan M., Gaotswe G., O’Connell J., Foley N., O’Neill L.A., Cody D., O’Shea D. (2014). The impact of childhood obesity on inflammation, innate immune cell frequency, and metabolic microRNA expression. J. Clin. Endocrinol. Metab..

[23] Atakan M.M., Koşar Ş.N., Güzel Y., Tin H.T., Yan X. (2021). The Role of Exercise, Diet, and Cytokines in Preventing Obesity and Improving Adipose Tissue. Nutrients.

[24] Brancaccio M., Mennitti C., Cesaro A., Fimiani F., Moscarella E., Caiazza M., Gragnano F., Ranieri A., D’Alicandro G., Tinto N. (2020). Dietary Thiols: A Potential Supporting Strategy against Oxidative Stress in Heart Failure and Muscular Damage during Sports Activity. Int. J. Environ. Res. Public Health.

[25] Calcaterra V., Vandoni M., Rossi V., Berardo C., Grazi R., Cordaro E., Tranfaglia V., Carnevale Pellino V., Cereda C., Zuccotti G. (2022). Use of Physical Activity and Exercise to Reduce Inflammation in Children and Adolescents with Obesity. Int. J. Environ. Res. Public Health.

[26] Salamt N., Muhajir M., Aminuddin A., Ugusman A. (2020). The effects of exercise on vascular markers and C-reactive protein among obese children and adolescents: An evidence-based review. Bosn. J. Basic Med. Sci..

[27] Wisse B.E. (2004). The inflammatory syndrome: The role of adipose tissue cytokines in metabolic disorders linked to obesity. J. Am. Soc. Nephrol..

[28] Utsal L., Tillmann V., Zilmer M., Mäestu J., Purge P., Jürimäe J., Saar M., Lätt E., Maasalu K., Jürimäe T. (2012). Elevated serum IL-6, IL-8, MCP-1, CRP, and IFN-γ levels in 10- to 11-year-old boys with increased BMI. Horm. Res. Paediatr..

[29] Liang Z., Pan F., Yang Z., Wang M., Hu C., Shi L., Ji Q., Liu L. (2021). Interleukin-9 deficiency affects lipopolysaccharide-induced macrophage-related oxidative stress and myocardial cell apoptosis via the Nrf2 pathway both in vivo and in vitro. Biofactors.

[30] Dorneles G.P., Haddada D.O., Fagundes D.O., Vargasa B.K., Kloecker A., Romão P.R.T., Peres A. (2016). High intensity interval exercise decreases IL-8 and enhances the immunomodulatory cytokine interleukin-10 in lean and overweight–obese individuals. Cytokine.

[31] Trøseid M., Lappegård K.T., Claudi T., Damås J.K., Mørkrid L., Brendberg R., Mollnes T.E. (2004). Exercise reduces plasma levels of the chemokines MCP-1 and IL-8 in subjects with the metabolic syndrome. Eur. Heart J..

[32] Petersen A.M.W., Pedersen B.K. (2005). The anti-inflammatory effect of exercise. J. Appl. Physiol..

[33] Luotola K. (2022). IL-1 Receptor Antagonist (IL-1Ra) Levels and Management of Metabolic Disorders. Nutrients.

[34] Aradillas-García C., Monreal-Escalante E., Vargas-Morales J.M., Alegría-Torres J., Rosales-Mendoza S., Terán-García M., Portales-Pérez D.P. (2021). Serum Il-17, obesity, and metabolic risk in mexican young adults. Rev. Salud Publica Nutr..

[35] Polak-Szczybyło E., Tabarkiewicz J. (2022). IL-17A, IL-17E and IL-17F as Potential Biomarkers for the Intensity of Low-Grade Inflammation and the Risk of Cardiovascular Diseases in Obese People. Nutrients.

[36] Dong-Hoon K., Sandoval D., Reed J.A., Matter E.K., Tolod E.G., Woods S.C., Seeley R.J. (2008). The role of GM-CSF in adipose tissue inflammation. Am. J. Physiol. Endocrinol. Metab..

[37] Lacy P. (2017). Chapter 12—Eosinophil Cytokines in Allergy. Cytokine Effector Function in Tissue.

[38] Suzuki Y., Yamaguchi M., Mori M., Sugimoto N., Suzukawa M., Iikura M., Nagase H., Ohta K. (2021). Eotaxin (CCL11) enhances mediator release from human basophils. Allergy.

[39] Moreno B., Hueso L., Ortega R., Benito E., Martínez-Hervas S., Peiro M., Civera M., Sanz M.-J., Piqueras l., Jose T. (2022). Real. Association of chemokines IP-10/CXCL10 and I-TAC/CXCL11 with insulin resistance and enhance leukocyte endothelial arrest in obesity. Microvasc. Res..

[40] Onogi Y., Wada T., Kamiya C., Inata K., Matsuzawa T., Inaba Y., Kimura K., Inoue H., Yamamoto S., Ishii Y. (2017). PDGFRβ Regulates Adipose Tissue Expansion and Glucose Metabolism via Vascular Remodeling in Diet-Induced Obesity. Diabetes.

[41] Pero R., Angrisano T., Brancaccio M., Falanga A., Lombardi L., Natale F., Laneri S., Lombardo B., Galdiero S., Scudiero O. (2019). Beta-defensins and analogs in Helicobacter pylori infections: mRNA expression levels, DNA methylation, and antibacterialactivity. PLoS ONE.

[42] Pero R., Brancaccio M., Laneri S., De Biasi M.G., Lombardo B., Scudiero O. (2019). A Novel View of Human Helicobacter pylori Infections: Interplay between Microbiota and Beta-Defensins. Biomolecules.

[43] Laneri S., Brancaccio M., Mennitti C., De Biasi M.G., Pero M.E., Pisanelli G., Scudiero O., Pero R. (2021). Antimicrobial Peptides and Physical Activity: A Great Hope against COVID 19. Microorganisms.

[44] Scudiero O., Brancaccio M., Mennitti C., Laneri S., Lombardo B., De Biasi M.G., De Gregorio E., Pagliuca C., Colicchio R., Salvatore P. (2020). Human Defensins: A Novel Approach in the Fight against Skin Colonizing *Staphylococcus aureus*. Antibiotics.

[45] Warburton D.E.R., Nicol C.W., Bredin S.S.D. (2006). Health benefits of physical activity: The evidence. CMAJ.

[46] Garber C.E. (2019). The Health Benefits of Exercise in Overweight and Obese Patients. Curr. Sports Med. Rep..

[47] Jamurtas A.Z., Stavropoulos-Kalinoglou A., Koutsias S., Koutedakis Y., Fatouros I. (2015). Adiponectin, resistin, and visfatin in childhood obesity and exercise. Pediatr. Exerc. Sci..

[48] Mărginean C.O., Meliţ L.E., Huțanu A., Ghiga D.V., Săsăran M.O. (2020). The adipokines and inflammatory status in the era of pediatric obesity. Cytokine.

[49] Cohen E., Margalit I., Shochat T., Goldberg E., Krause I. (2021). Markers of Chronic Inflammation in Overweight and Obese Individuals and the Role of Gender: A Cross-Sectional Study of a Large Cohort. J. Inflamm. Res..

[50] https://www.cdc.gov/healthyweight/assessing/bmi/childrens_bmi/about_childrens_bmi.html.

[51] Thomas-Eapen N. (2021). Childhood Obesity. Prim. Care.

[52] Mathur N., Pedersen B.K. (2008). Exercise as a mean to control low-grade systemic inflammation. Mediat. Inflamm..

[53] Gonzalo-Encabo P., Maldonado G., Valadés D., Ferragut C., Pérez-López A. (2021). The Role of Exercise Training on Low-Grade Systemic Inflammation in Adults with Overweight and Obesity: A Systematic Review. Int. J. Environ. Res. Public Health.

[54] Tagi V.M., Giannini C., Chiarelli F. (2019). Insulin Resistance in Children. Front. Endocrinol. Sec. Pediatr. Endocrinol..

[55] Richter E.A., Hargreaves M. (2013). Exercise, GLUT4, and skeletal muscle glucose uptake. Physiol. Rev..

[56] El-Mikkawy D.M.E., El-Sadek M.A., El-Badawy M.A., Samaha D. (2020). Circulating level of interleukin-6 in relation to body mass indices and lipid profile in Egyptian adults with overweight and obesity. Egypt Rheumatol. Rehabil..

[57] Wang T., Qiang Jiang C., Xu L., Sen Zhang W., Zhu F., Li Jin Y., Thomas G.N., Keung Cheng K., Hing Lam T. (2018). White blood cell count and all-cause and cause-specific mortality in the Guangzhou biobank cohort study. BMC Public Health.

[58] Johannsen N.M., Swift D.L., Johnson W.D., Dixit V.D., Earnest C.P., Blair S.N., Church T.S. (2012). Effect of Different Doses of Aerobic Exercise on Total White Blood Cell (WBC) and WBC Subfraction Number in Postmenopausal Women: Results from Drew. PLoS ONE.

